# Characteristics of occupational musculoskeletal disorders of five sectors in service industry between 2004 and 2013

**DOI:** 10.1186/s40557-017-0198-4

**Published:** 2017-09-19

**Authors:** Hyun-Woo Choi, Young-Ki Kim, Dong-Mug Kang, Jong-Eun Kim, Bo-Young Jang

**Affiliations:** 1 Department of Occupational & Environmental Medicine, Pusan National University Yangsan Hospital, (626-770) 20, Geumo-ro, Mulgeum-eup, Yangsan-si, Gyeongsangnam-do South Korea; 2 Department of Preventive and Occupational Medicine, School of Medicine, Pusan National University, Busan, South Korea; 3 Institute of Environmental health research, Pusan National University, Yangsan, South Korea

**Keywords:** Industrial injury, Musculoskeletal disorders, Work related musculoskeletal disorders, Service industry

## Abstract

**Background:**

**‘**Work related musculoskeletal disorders (WRMSDs)’ have been mostly reported in the manufacturing industry but recently the occurrence of industrial injuries has been constantly increasing in the service industry. This research is going to analyze the data about workers’ compensation for WRMSDs in five different service sectors and identify characteristics of occupations with the highest approved occupations.

**Methods:**

According to the data released from the Korea Worker’s Compensation & Welfare Service, the overview of 12,730 cases of workers’ compensation for WRMSDs in five service sectors from 2004 to 2013 is going to be analyzed and the source data is going to be classified by the Korean Standard Classification of Occupations to select the top five occupations that have the highest number of approval.

**Results:**

After selecting each five occupations from the service sector that have work related musculoskeletal disorders, the result showed that the occupation with the highest number of approval in the health and social care sector were the early childhood educators, cooks in the school canteens in education services sector, garbage collectors in the sanitation and similar services sector, deliverymen in wholesale and retail, consumer goods repair and building cleaners in general management businesses such as those in building maintenance. The major event observed in the top five occupations was the overexertion and reaction as a cause of WRMSDs. The day when the WRMSDs mostly occurred was on Monday and the most likely time was 10 am. The median days away from work and lost working days are 29–90 days and 0–50 days respectively. The difference in each occupation was observed in year of service, age, and gender.

**Conclusions:**

83.21% of the approved cases of workers’ compensation for WRMSDs occurred in the top 25 occupations in all of the five service sectors, which meant that the approval of workers’ compensation is concentrated in specific occupations. This research is going to suggest preventive measures for work related musculoskeletal disorders in the service industry and to help prioritize the preventive measures.

**Trial registration:**

Not applicable.

## Background

Work related musculoskeletal disorders (WRMSDs) show symptoms of constant pain of muscles, tendons, nerves and blood vessels, which is mostly caused by repetitive motion or overexertion. This accounted for 69.7% of the total occupational disorders that occurred in South Korea in 2013, which in turn had the highest proportion among occupational disorders [1, 2].

The statistics reported from the Ministry of Employment and Labor showed that the occurrence of WRMSDs has been constantly increasing until 2003 while most of the incidents occurred in the manufacturing industry, especially in shipbuilding companies and automobile companies [3]. After 2003 the recent change of the industry structure in South Korea from the manufacturing industry into the service industry affected WRMSDs causing an increase in the service industry [3]. The proportion of service industry accounts for 59% of all business units and 42% of the number of employees (2009). The proportion of all the workers was 35% (33,961 cases) of the total occupational injury cases, which was up from 23% (19,342 cases) in 2001, exceeding the proportion of manufacturing industry of 34% (32,997) as of 2009 [4–7]. Also, depending on the occupation, the WRMSDs account for 46% in manufacturing industry in 2013 followed by 37% in the service industry [2]. As of 2003, the WRMSDs has sustained increases in the service industry while decreases were noted in the manufacturing industry as of 2004 [8].

Referring to the statistical data from developed countries, the Bureau of Labor Statistics (BLS) of USA has analyzed data to identify risk factors and the actual condition of WRMSDs since the 1980s [9]. It was reported that the overall number of workers with WRMSDs have been decreasing and the disorders have occurred greater in the service, wholesale, retail, and transportation sectors than in the manufacturing industry [9]. Likewise, the increasing trend in the service industry might be observed in South Korea.

Despite this current condition, there has been a lack of research on conditions and characteristics of WRMSDs in the service industry. The literature reviews about the characteristics of WRMSDs are limited to a certain industry such as manufacturing and construction or of certain regions [10]; therefore, it is difficult to compare with various occupations.

Accordingly, this research is going to analyze the data provided about cases accredited as approved workers’ compensation for WRMSDs in the top five occupations that account for the majority of service industry from 2004 to 2013 in order to figure out the occupations with the highest number of approval, the characteristics of WRMSDs in the service industry and finally to set the priority of preventive measures.

## Methods

The occupations are selected based on the approved workers’ compensation data from the Korea Workers’ Compensation & Welfare Service (Kcomwel), which are the 12,730 cases of workers suffering from WRMSDs in five major service sectors – Health and social care, Education services, Sanitation and similar services, Wholesale and retail, consumer goods repair, General management business such as building management. The limited data was provided by Kcomwel as part of research, the development of hazard investigation guide for Korean Safety & Health Agency (2014).

Then, the occupations were classified into groups and the top five occupations with the highest number of approval cases in each group were selected as an object of research. The classification standard of occupations varied by regions, therefore the source data was reclassified by the Korean Standard Classification of Occupations from Statistics Korea. First of all, the classification system of the source data and the size of the occupations – large, medium, and small - were considered as the first criteria, then the outline of WRMSDs was considered for the final decision of classification. In other words, if the size of the occupation corresponded to the outline of the industrial disorders then the occupation would be classified by the standard classification of the occupation. If the occupation was not listed in the standard classification of occupation, referred to as being unable to be classified or the classification system of the source data did not correspond to the outline of the industrial accidents, then it was classified to the closest one by comparing the outline of the occupation and actual WRMSDs cases one by one.

The characteristics of occupations with the highest number of approval were categorized by the types of disorders, the day it happened, median days away from work, year of service, age group, gender, lost working days and time when the injury occurred. The characteristics were then listed by the top five occupations in five major service sectors and the top 25 occupations overall.

## Results

### Demographical characteristics of workers with WRMSDs in five major service sectors

The proportion for men (60.82%) was higher than women (39.18%). Workers in the age groups of 30–39 and 40–49 accounted for 29.67% and 26.35% respectively. According to the type of disorders, accidental lower back pain and non-accidental lower back pain were frequently observed in 57.18% and 22.58% of the employees respectively (Table 1).

**Table 1:** Demographic characteristics to approval data of workers’ compensation for WRMSDs of five sectors in service industry

Variables		Number	Percentage
Gender	Male	7742	60.82
	Female	4988	39.18
Age(year)	18 ~ 29	1544	12.13
	30 ~ 39	3777	29.67
	40 ~ 49	3354	26.35
	50 ~ 59	2783	21.86
	≥ 60	1272	9.99
Disorder^a^	Accidental low back pain	7279	57.18
	Non-accidental low back pain	2874	22.58
	Musculoskeletal burdened works	2410	18.93
	Carpal tunnel syndrome	167	1.31

^a^The data was classified based on WRMSDs classification code as authorization of workers’ compensation data of Korea Workers’ Compensation & Welfare Service (Kcomwel)

### Selection of the occupations with the highest number of approval for WRMSDs in the major five service sectors

The top five occupations from the five major service sectors were selected (Table 2). 2681 workers suffered from industrial disorders in the health and social care sector from 2004 to 2013. Early childhood educators were the highest number of approved occupation followed by health care assistants working in hospitals and cooks working in hospitals, social welfare facilities and kindergarten. Workers in the education services sector had 1126 WRMSDs. The occupation with the highest number of approval in the education service sector was cooks in the school canteens accounting for 73.27% of the total cases. Workers in the sanitation and similar services sector had 1423 WRMSDs while a garbage collector was listed as the top followed by a street sweeper and a recycling collector. Then, a construction laborer in charge of street and drain maintenance followed. The occupations that had the highest number of WRMSDs in the five service sectors included wholesale, retail and consumer goods repair, which accounted for 5891 cases. The occupation with the highest number of approval was transport laborers who were general goods, liquor and beverage deliverymen in supermarket, department stores and markets. This was followed by storage laborers who usually load and unload goods at warehouses, supermarkets and department stores. Shop salespersons are referred to as persons that work in supermarkets, department stores, markets and managers in charge of sales, transportation, administration, management support, planning and promotions, marketing, security in wholesale and retail sector. Workers in the general management businesses such as building management had 1609 WRMSDs and the occupation with the highest number of approval included building structure cleaners followed by apartment security guards, building supervisors, building caretakers and electrical equipment installers and repairers.

**Table 2:** Top five occupations of the highest number of approval for WRMSDs of each five sectors in service industry

Sectors(N)	Occupations	Number	Percentage
Health and social care (2681)	Early childhood educators	487	18.16
	Health care assistants	394	14.70
	Cooks^a^	302	11.26
	Social welfare managers^b^	223	8.32
	Nursing professionals	200	7.46
Education services (1126)	Cooks^c^	825	73.27
	Office clerks	51	4.53
	Teaching professionals	38	3.75
	Teaching professionals not elsewhere classified^d^	21	1.87
	Early childhood educators	20	1.78
Sanitation and similar services (1423)	Garbage collectors	466	32.75
	Sweepers	265	18.62
	Recycling collectors	119	8.36
	Construction labourers	109	7.66
	Forestry labourers^e^	68	4.78
Wholesale, retail and consumer goods repair(5891)	Transport labourers^f^	1735	29.45
	Storage labourers^g^	837	14.21
	Shop salespersons	833	14.14
	Retail and wholesale trade managers	526	8.93
	Agricultural and sea food salesworkers	368	6.25
General management business such as building management (1609)	Building structure cleaners	649	40.34
	Domestic housekeepers	323	20.07
	Building supervisors	204	12.68
	Building caretakers	65	4.04
	Electrical equipment installers and repairers	42	2.61

^a^Cooks in health and social care: mainly worked for kindergarten, hospital and social welfare facilities

^b^Social welfare managers: mainly it refers to home visit care worker

^c^Cooks in education service: worked for school meals

^d^Teaching professionals not elsewhereclassified: worked as assistants in science lab, art room, computer classroom and special-education school

^e^Forestry labourers: engaged in flower beds, tree-lined road and park maintenance

^f^Transport labourers: general goods, liquor and beverage deliveryman in Mart, department stores and markets

^g^Storage labourers: engaged in loading and unloading at warehouse, Mart, department stores and markets

Then the top 25 occupations from the five major service sectors with the highest number of approval with similarities were all grouped together and selected for the study (Table 3). The top 25 occupations accounted for 83.21% of the overall five major service sectors while the top 10 occupations accounted for 60.84%. Of the all occupations, those that are counted duplicate in both five-occupation sectors were cooks and early childhood educators. By adding up the number of workers counted as duplicates, there was a slight difference in the rank. Newly added occupations included personal care workers, home-based personal care workers, shop supervisors, nursing professionals, and gas station workers. Personal care workers are referred to as workers working in social service centers such as welfare centers or sanatorium while home-based personal care workers are in charge of the home visiting volunteer services and domestic help services.

**Table 3:** Top twenty-five occupations of the highest number of approval for WRMSDs of all five sectors in service industry

Occupations	Number	Percentage
Transport labourers	1742	13.67
Cooks	1208	9.48
Storage labourers	886	6.95
Shop salespersons	833	6.54
Building structure cleaners	740	5.81
Retail and wholesale trade managers	543	4.26
Early childhood educators	506	3.97
Garbage collectors	505	3.97
Health care assistants	408	3.20
Office clerks	381	2.99
Agricultural and sea food sales workers	369	2.90
Domestic housekeepers	325	2.55
Sweepers	269	2.11
Social welfare managers	233	1.83
Building supervisors	204	1.60
Recycling collectors	200	1.57
Personal care workers in health services^a^	172	1.35
Home-based personal care workers^b^	169	1.33
Shop supervisors	166	1.30
Paramedical practitioners	162	1.27
Nursing professionals	143	1.12
Construction labourers	131	1.03
Gas station workers	126	0.99
Building caretakers	95	0.75
Electrical equipment installers and repairers	85	0.67
Total	10,601	83.21

^a^Personal care workers in health services: worked in social services (welfare centers, sanatorium)

^b^Home-based personal care workers: domestic helpers for the elderly

### Characteristics of the top 25 occupations with WRMSDs in the five major service sectors

The characteristics are categorized by cause, day, days away from work for treatment, year of service, age, gender, lost working days and the time when the injury occurred. Then occupations with the highest number of approval were analyzed (Table 4). The leading major causes of disorders observed in the top 25 occupations were overexertion and repetitive motions except for the option, not applicable. The day when the industrial accident frequently happened was Monday and the time at which it occurred was 10:00 a.m. The days away from work for treatment were between 29 and 90 days and the amount of lost work days were between 0 and 50 days.

**Table 4:** Common Characteristics of the top 25 occupations with WRMSDs in the five major service sectors

Variables		Number	Percentage
The leading major causes of disorders	not applicable	5243	71.20
	overexertion	1631	22.15
	repetitive motions	311	4.22
	the others	179	2.43
The day when the industrial accident frequently happened	Monday	1549	21.04
	Tuesday	1145	15.55
	Wednesday	1121	15.22
	Thursday	1216	16.51
	Friday	1316	17.87
	Saturday	710	9.64
	Sunday	307	4.17
The time at which accident occurred	10:00	1471	19.98
	09:00	968	13.14
	11:00	879	11.94
	the others	4046	54.94
The days away from work for treatment	29-90 days	3315	45.02
	91–180 days	1768	24.01
	the others	2281	30.97
The amount of lost work days	0-50 days	2549	34.61
	51–100 days	2111	28.67
	the others	2704	36.72

Data including missing values were excluded

**Fig. 1:**
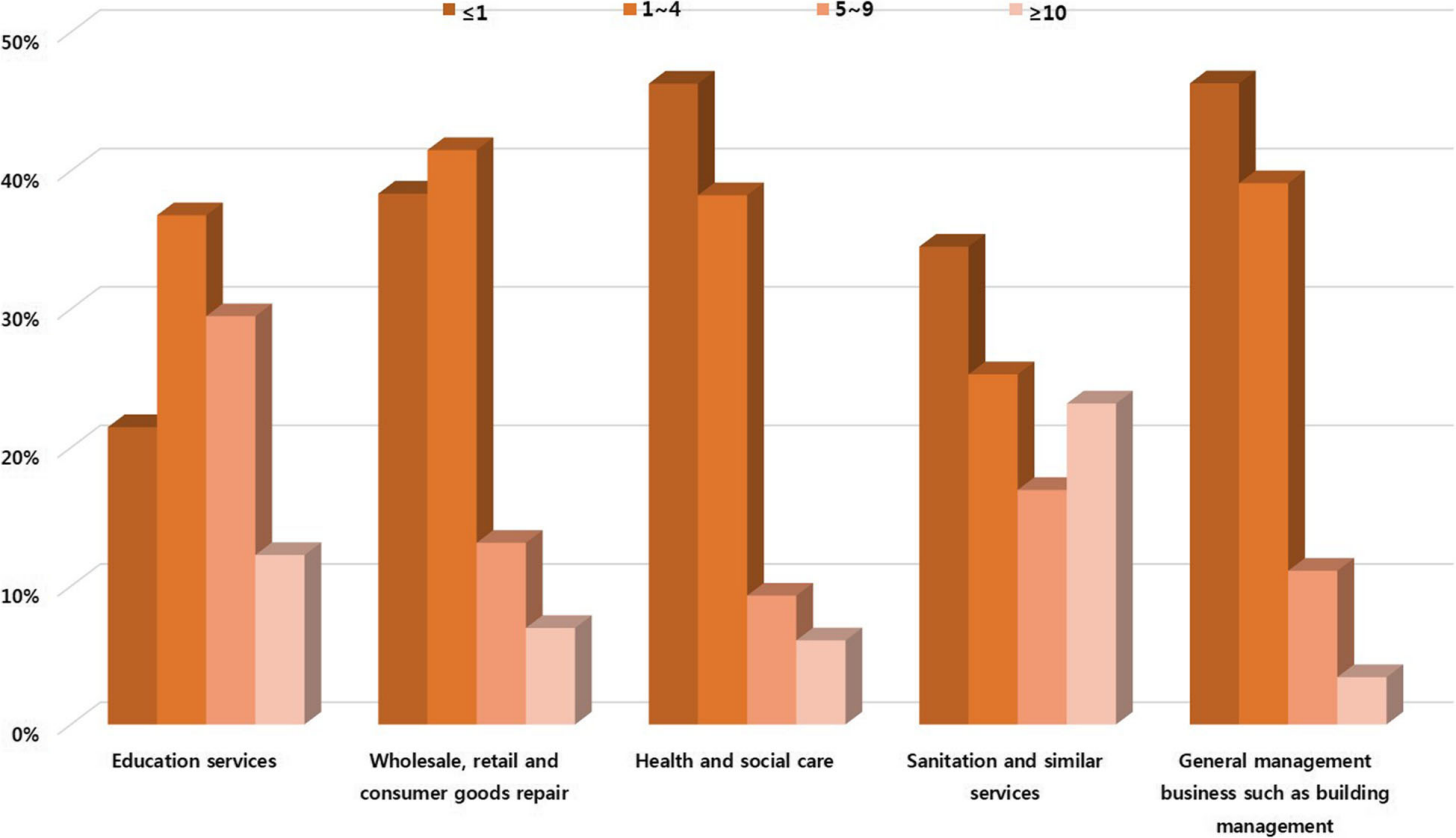
Different characteristics of the top 25 (the highest number of approval) occupations with work related musculoskeletal disorders in each five major service sectors; Year of service(years)

**Fig. 2:**
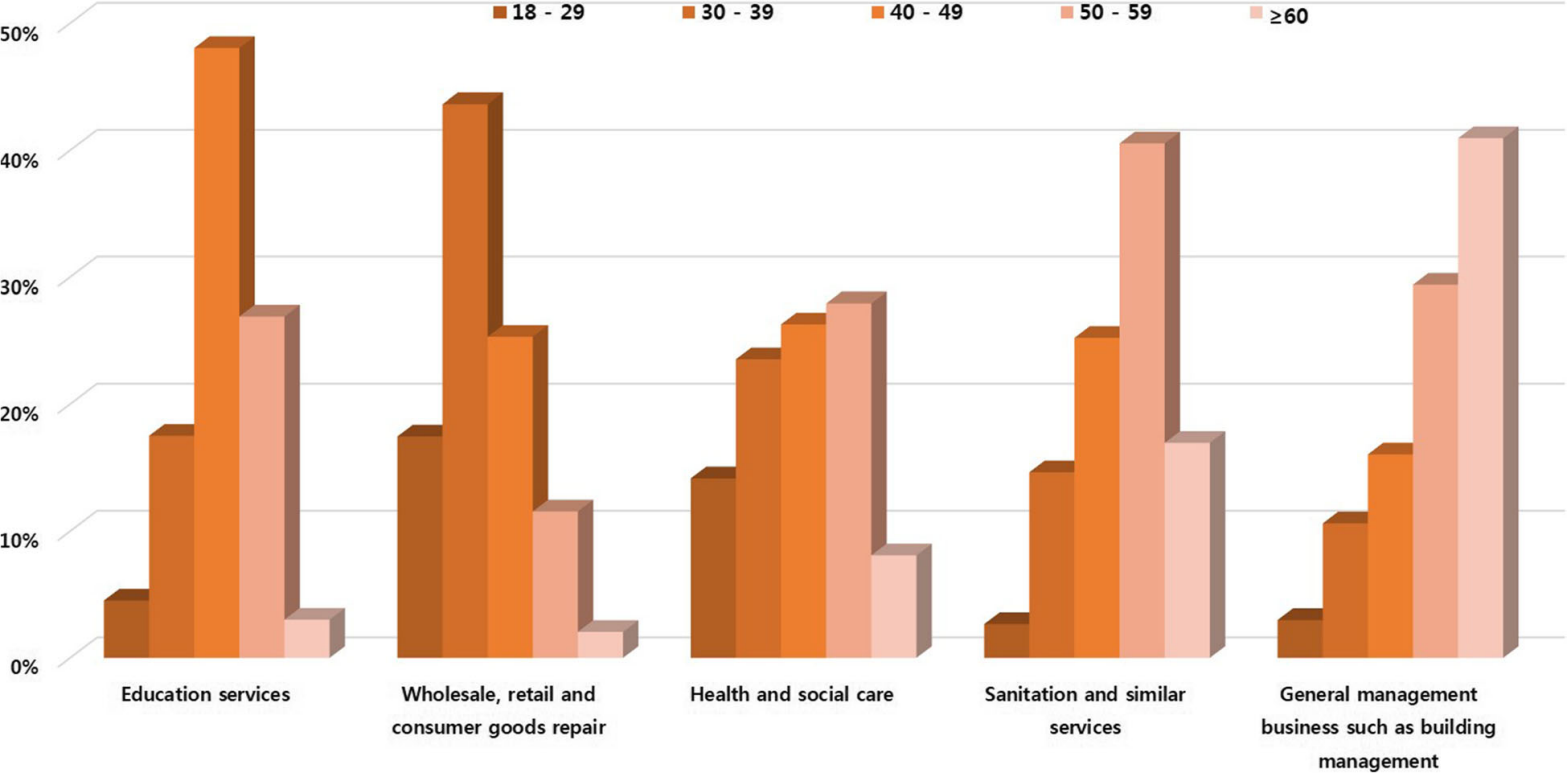
Different characteristics of the top 25 (the highest number of approval) occupations with work related musculoskeletal disorders in each five major service sectors; age(years)

**Fig. 3:**
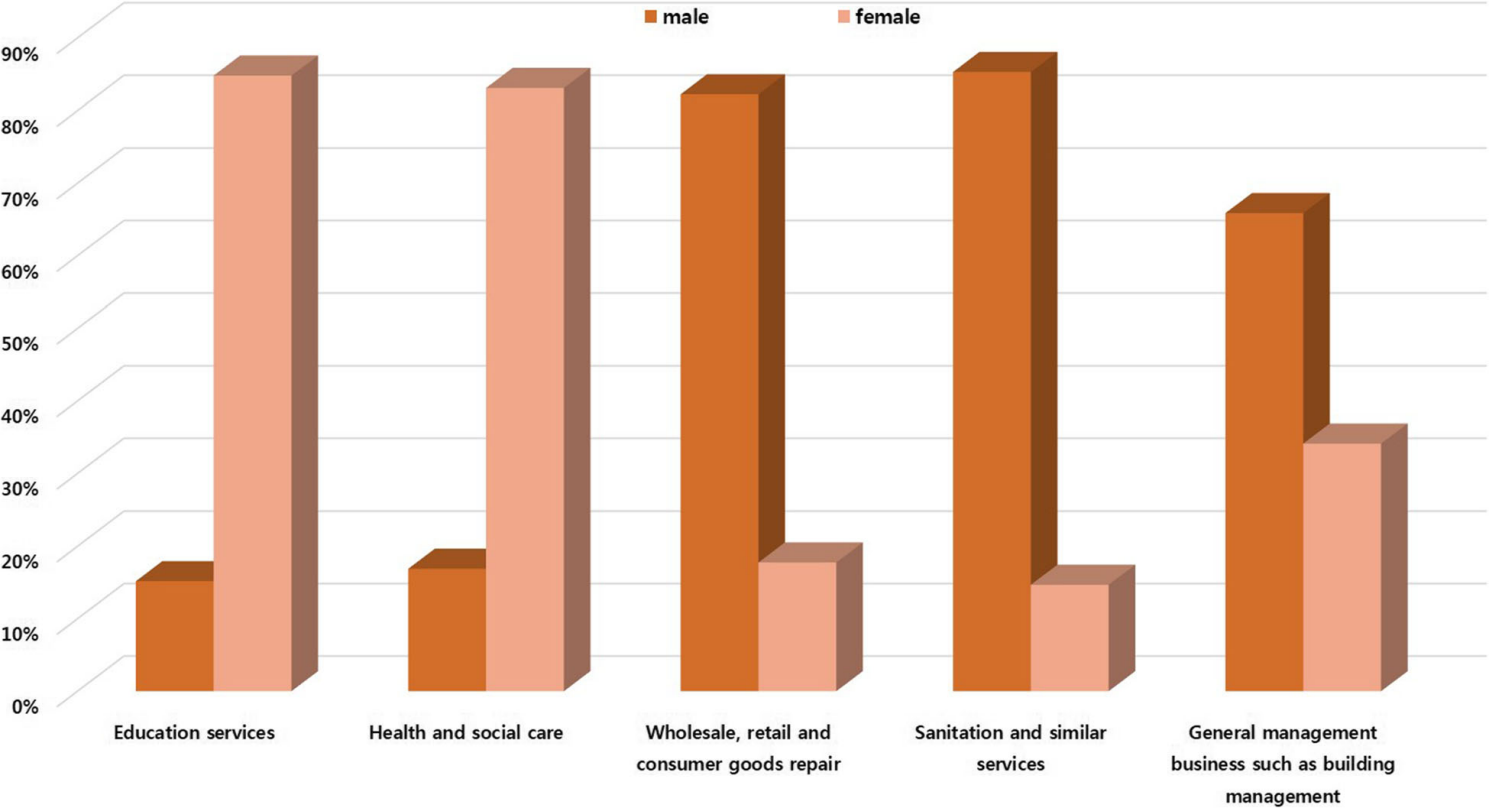
Different characteristics of the top 25 (the highest number of approval) occupations with work related musculoskeletal disorders in each five major service sectors; sex

The differences in each occupation were the year of service, age and gender (Figs. 1-3). The occupations with the year of service less than 1 year with the highest proportion were found in health and social care, sanitation and similar services sectors and general management businesses such as building management. The occupations that had the highest proportion by year of service were found in education services averaging between 1 and 4 years along with the wholesale and retail, consumer goods repair. However, the occupations with a relatively high proportion of long-term employees over five years were education services and sanitation and similar services (Fig. 1). The occupations that had the highest proportion in the age group of 30–39 were found in wholesale, retail and consumer goods repair sector. The occupations with the highest proportion in the age group of 40–49 were in the education services sector and in the age group of over 50 are in the sanitation and similar services, health and social care, general management business such as building management (Fig. 2). By gender, men had the highest proportion in wholesale, retail, consumer products, sanitation and similar services and general management businesses such as building management than women. Women had the highest proportion in health and social assistance and education services sector than men (Fig. 3).

## Discussion

Among the top five service sectors with WRMSDs, the top 25 occupations accounted for 83.21%, which means that the approval of workers’ compensation is concentrated in specific occupations. Chan-Young Yoo et al. (2009) reported that 10 occupations with the highest WRMSDs proportion from a total 62 occupations accounted for 63.7–64.9% based on the Industrial Accident Compensation Insurance Act. This proved that the WRMSDs have occurred intensively depending on the characteristics of occupations and they also pointed out that the characteristics of disorder occurrence should be analyzed in detail by occupation and size [11].

By comparing the characteristics of WRMSDs in the top 25 occupations of the five major service sectors with those of manufacturing industry, which has the most frequent occurrence of WRMSDs in Korea, men in the manufacturing sector (machinery manufacturing for analytical transportation) accounted for 98.9% and workers in the 30–39 and 40–49 year age groups had the highest number of approval [12]. Workers between 10 and 20 years of service had the highest proportion with most of the incidents occurring between 10:00 am - 12:00 pm. The most common cause was overexertion [12]. The cause and time of incidents with the highest proportion in the major five service sectors coincided with those of the manufacturing industry but the years of service, age and gender showed differences between two industries [12]. According to the year of service, occupations in health and social care, sanitation and similar services sectors and general management businesses such as building management showed the highest number of approval when the year of service was less than 1 year while the manufacturing industry showed 10–20 years [12]. This was because workers in manufacturing industry are more likely to experience long-term service and have higher risk factors of industrial accident due to the accumulated exposure of danger. Service sector workers on the other hand have higher risk factors of industrial accident due to inexperience or negligence. By age group, manufacturing workers are likely to start work at a younger age and have long-term service while service workers especially in sanitation and similar services sector (garbage collectors), health and social care (early childhood educators, nursing professionals), general management business such as building management (building cleaners), start work at a relatively older age. By gender, there’s no significant difference in service industry against manufacturing industry. This might be due to the higher number of females in the service industry than those in the manufacturing industry.

The results also revealed that the WRMSDs in the top 25 occupations had the highest occurrence on Monday when the new week begins and at 9:00, 10:00, 11:00 am when work begins. It is assumed that the incidents are likely to occur when workers are not accustomed to their duties. Therefore, the workplace need preventive measures on the days and times just noted. For the year of service, some occupations showed the highest rate less than 1 year and some between 1 to 4 years. In addition, the occupations with the highest number varied by age group, which means that each of the occupations need customized educational programmes depending on their characteristics.

To effectively prevent the WRMSDs, high risk groups in terms of the most frequently occurred needs to be set and the number of researchers, budget to be invested in occupational disorders prevention and annual target should be considered. Also, with focus on the occupations with higher number of approval for WRMSDs, preventive measure programs should be prepared and applied in the workplace so that the program could be implemented autonomously. In the U.S, the number of WRMSDs has been constantly decreasing by the implementation of human engineering preventive strategies since 1994 and its National Institute of Occupational Safety and Health (NIOSH) has regulated the MSD preventive measures and safety guidelines. For example, there are Home health care MSD (musculoskeletal disorders) prevention: How to prevent musculoskeletal disorders (2012), Preventing slips, trips, and falls in wholesale and retail establishments (2012). The major occupations include home health care, the soft drink beverage delivery industry, wholesale and retail, patient handling and home building. Disorder and injury prevention strategies for the mentioned occupations in the service industry have been set, conducted and examined [13]. For the home health care MSD prevention, the number of cases in home health care occupations have twice higher than those of full-time workers in the manufacturing industry and half of the industrial accident cases accounted for WRMSDs, therefore, suggesting strategies to safely take care of patients while preventing the disorders [14].

Meanwhile, Occupational Safety and Administration (OSHA) provides human engineering guidelines to prevent WRMSDs in retail grocery stores, nursing homes and meatpacking occupations. For example, there are Guidelines for retail grocery stores (2013), Guidelines for nursing home (2009), Safety and health guide for the meatpacking industry (1988) [15]. In addition, the National Occupational Research Agenda (NORA) is a partnership program to stimulate innovative research and improve the workplace as a work related research system from U.S. government, which has been a national research framework together with NIOSH since it has been reported in 1996. Experts from various fields have been cooperating to identify safety and health issues in the workplace and especially for the service industry, they have categorized them into automobile repair, construction services, education services, hotel and inn services, public administration, recreation and entertainment, restaurant service or food suppliers, telecommunication, part-time services, garbage collection and disposal, hair and nail salon to set strategy goals, monitoring the goals, mid-term goals, research objectives, conducting objectives of occupational safety and health then monitor them on a regular basis.

While in Europe, they selected occupations with the highest number of approval for the WRMSDs due to repetitive motion such as workers in the textile manufacturing industry and introduced disorder preventive cases from European countries while sustaining their research on occupations with risk factors of the disorder [16]. Considering those programs abroad, our country, South Korea, needs to edit and improve specific preventive strategies depending on each occupations and examine regularly whether the preventive program is followed.

This research has some limitations; firstly, the statistic data about WRMSDs only included the cases that were accredited as an workers’ compensation, therefore, the cases that were not accredited such as the concealment of industrial accidents, direct compensation in company, individual treatment, company without industrial accident insurance could not be included. However, data from administrative systems are incomplete because not all WRMSDs are compensable [17], workers’ compensation data is one of the method used to surveillance souces for WRMSDs [18].

Also, the personal variations such as workers’ physical attributes (height, weight, etc), health conditions (medical history), lifestyles (smoking, drinking, working out, leisure time activity, etc.), social psychological factors (company culture, job stress, etc.) could not be considered. Therefore, further research considering those variations is recommended.

Additionally, categorizing occupations based on the Korean Standard Classification of Occupations had some difficulties. There were occupations that were not classified in the data from the Korea Workers’ Compensation & Welfare Service and the existing classification system did not correspond with the data from the Korean Standard Classification of Occupations, which required us to compare every cases individually to find out the closest occupation group, therefore, led to a possibility of misclassification. We tried to reduce the error by letting one person take a charge of classification. For the future study, the local center needs to unify the classification system and follow the Korean Standard Classification of Occupations.

Despite those limitations, this research found the occupations with the highest number of approval and the characteristics of WRMSDs in five major service sectors. However, further research on year of service, age, and gender is needed since those characteristics showed differences in each service sectors. To be more precise, the analysis should be conducted based on unified WRMSDs occurrence statistical data released according to the Korean Standard Classification of Occupations. This research has looked at the characteristics of occupations with the highest number of approval for WRMSDs in five major service sectors, which in turn is expected to help prioritize the preventive strategies in service industry.

## Conclusions

In summary, the approval of workers’ compensation is concentrated in specific occupations. This research identified the characteristics of the top 25 occupations with the highest number of approval for WRMSDs in five service sectors; therefore, it will help prioritize the preventive strategies of disorder in service industry.

## Abbreviations

Kcomwel: Korea Workers’ Compensation & Welfare Service
MSD: Musculoskeletal disorders
NIOSH: National institute of occupational safety and health
NORA: National Occupational Research Agenda
OSHA: Occupational Safety and Administration
WRMSDs: Work related musculoskeletal disorders
